# Pathway Analysis Using Information from Allele-Specific Gene Methylation in Genome-Wide Association Studies for Bipolar Disorder

**DOI:** 10.1371/journal.pone.0053092

**Published:** 2013-01-09

**Authors:** Li-Chung Chuang, Chung-Feng Kao, Wei-Liang Shih, Po-Hsiu Kuo

**Affiliations:** 1 Department of Public Health & Institute of Epidemiology and Preventive Medicine, College of Public Health, National Taiwan University, Taipei, Taiwan; 2 Department of Nursing, Cardinal Tien College of Healthcare & Management, I-Lan, Taiwan; 3 Research Center for Genes, Environment and Human Health, National Taiwan University, Taipei, Taiwan; University of Iowa Hospitals & Clinics, United States of America

## Abstract

Bipolar disorder (BPD) is a complex psychiatric trait with high heritability. Despite efforts through conducting genome-wide association (GWA) studies, the success of identifying susceptibility loci for BPD has been limited, which is partially attributed to the complex nature of its pathogenesis. Pathway-based analytic strategy is a powerful tool to explore joint effects of gene sets within specific biological pathways. Additionally, to incorporate other aspects of genomic data into pathway analysis may further enhance our understanding for the underlying mechanisms for BPD. Patterns of DNA methylation play important roles in regulating gene expression and function. A commonly observed phenomenon, allele-specific methylation (ASM) describes the associations between genetic variants and DNA methylation patterns. The present study aimed to identify biological pathways that are involve in the pathogenesis of BPD while incorporating brain specific ASM information in pathway analysis using two large-scale GWA datasets in Caucasian populations. A weighting scheme was adopted to take ASM information into consideration for each pathway. After multiple testing corrections, we identified 88 and 15 enriched pathways for their biological relevance for BPD in the Genetic Association Information Network (GAIN) and the Wellcome Trust Case Control Consortium dataset, respectively. Many of these pathways were significant only when applying the weighting scheme. Three ion channel related pathways were consistently identified in both datasets. Results in the GAIN dataset also suggest for the roles of extracellular matrix in brain for BPD. Findings from Gene Ontology (GO) analysis exhibited functional enrichment among genes of non-GO pathways in activity of gated channel, transporter, and neurotransmitter receptor. We demonstrated that integrating different data sources with pathway analysis provides an avenue to identify promising and novel biological pathways for exploring the underlying molecular mechanisms for bipolar disorder. Further basic research can be conducted to target the biological mechanisms for the identified genes and pathways.

## Introduction

Bipolar disorder (BPD) is a severe and complex psychiatric disorder, with high heritability around 0.6 to 0.7 [1], [2]. Prior individual linkage studies and meta-analyses suggested a number of susceptible regions in human genome for the risk of developing BPD. However, most of these findings are inconsistent and rarely pointed to specific chromosomal locations for replication [3]. Recently, large scale genome-wide association (GWA) studies, which scanned half or a million single nucleotide polymorphisms (SNPs), were frequently employed. Although the GWA studies were anticipated to provide comprehensive genetic information for complex traits, previous GWA studies for BPD reported limited numbers of susceptible loci with small effect size. The odds ratios of significant findings in GWA studies for BPD in populations of European, American and Han Chinese were between 1.2 and 2.0 [4], [5], [6], [7], which are in accordance with the observations from other GWA studies in complex traits [8]. In addition, the reported associated variants from GWA studies often explain a small proportion of heritability for complex traits, a so called ‘missing heritability’ phenomenon [9]. Missing heritability may be owing to lack of power to detect common variants with very small effect, not including rare variants for their effects in whole-genomic array, or not considering other genomic mechanisms, such as complex gene-gene interaction and epigenetic influences [10].

In most of the genetic studies, a commonly applied strategy is to analyze single markers or specific haplotypes for their associations with disease of interests. This often produces limited success in identifying putative loci for BPD, especially for variants with small to moderate effect. The genetic causes of BPD are likely involved with a large collection of genetic variants in certain biological pathways to jointly exhibit their effects for the trait. Therefore, pathway-based approach becomes a useful and complementary method in addition to single locus analyses. A pathway is considered as a specific gene set that is defined according to certain biological function or process. Analyzing GWA dataset with pathway-based approach could provide integrating information of multiple loci with similar physiological functions to bring biological insights into the mechanisms of BPD. Previously, pathway analysis has been successfully conducted using GWA datasets for several complex traits, such as schizophrenia, major depressive disorder, and breast cancer [11], [12], [13] to reveal important biological mechanisms underlying the diseases.

To perform pathway analysis using GWA dataset, an important first step is to extract SNPs information for each gene region. A commonly adopted method is to select the most significant SNP within a gene region to represent the gene [14]. Often, tens to hundreds of common SNPs are found in a typical gene region. Only a few of them are functional variants, thus, SNP with the maximum statistic may lack direct biological meaning and connection with the trait of interest. Nevertheless, non-structured variants may still regulate gene functions through other mechanisms. Epigenetic changes, one of regulatory mechanisms, can modify gene activity or gene expression without altering the genomic structure, including stable DNA methylation, post-translational modifications of histone proteins, and non-coding RNA [15]. Among these epigenetic modifications, changes of DNA methylation patterns at CpG sites are considered heritable and may play important roles in regulating gene functions [16], [17]. A pilot study of the Human Epigenome Project reported that more than 50% of CpG sites have greater than 50% variation within region of the major histocompatibility complex [18]. Compelling evidence also reveals a commonly observed phenomenon called allele-specific methylation (ASM) to describe different status of DNA methylation of a nearby CpG site by the two alleles presenting in a cell [19].

The profiles of DNA methylation are dynamic and tissue specific. Using human adult cerebellum samples, Zhang and colleagues (2010) conducted whole genome genetic polymorphisms and methylation quantitative association analysis to identify SNPs that regulate DNA methylation of CpG sites through *cis-* or *trans-* regulation [20]. Their findings documented that variation in genetic polymorphism affects the degree of DNA methylation in coding or non-coding region of specific genes. A considerable proportion of CpG sites were regulated by specific genetic variants distributed in the whole genome. Incorporating the ASM information into pathway-based analysis using GWA dataset may provide a new avenue to search for important biological pathways and to investigate the underlying pathogenesis of BPD.

The present study aimed to integrate brain-specific ASM information into whole genome genotyping data to identify important pathways for bipolar disorder. We used two GWA datasets of BPD with relatively large samples in Caucasian populations, the Wellcome Trust Case Control Consortium (WTCCC) and the Genetic Association Information Network (GAIN). The list of brain-specific ASM was obtained from the Zhang's (2010) study. We applied comprehensive pathway based statistical approaches with novel weighting scheme to incorporate the impacts of ASM to evaluate the enrichment of annotated pathways for BPD. The present study successfully identified significant and novel pathways for BPD. Our strategy to explore potential mechanisms for BPD through integrating information from different genomic aspects can be well applied to other complex traits.

## Materials and Methods

### Genome-wide association (GWA) datasets

In the current study, two GWA datasets of BPD in the Caucasian populations were used, the WTCCC [4] and the GAIN data [21]. We used these two individual GWA datasets to search for consistent pathway findings for BPD. The details of subject enrollment and genotyping of the two GWA studies were provided in their primary articles. In brief, all participants in the WTCCC were self-identified as white Europeans who lived in the United Kingdom. These included 1,868 subjects with BPD and 2,938 healthy subjects from the 1958 British Birth Cohort or United Kingdom Blood Donors. In the GAIN dataset, individuals were Americans with European ancestry, including 1,001 cases of BPD and 1,034 controls. The genotyping platform was Affymetrix GeneChip Human Mapping 500K Array and Affymetrix Genome-Wide Human SNP Array 6.0 for the WTCCC and GAIN, respectively. After quality control procedures implemented, a total of 485,263 (WTCCC) and 698,227 (GAIN) autosomal SNPs were retained in the following analyses [4], [21]. All single marker association analyses with additive model were conducted using PLINK versions 1.07 [22].

### Brain specific allele-specific gene methylation (ASM) list and computing gene-wise statistic values

**Figure 1:**
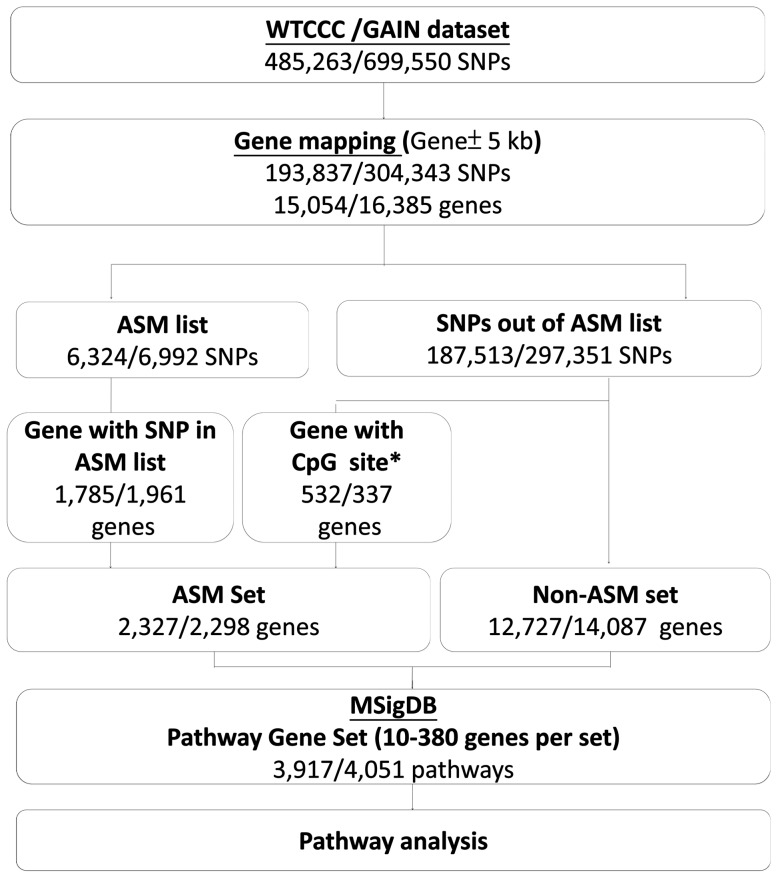
The summary description of present pathway-based method. *Gene with CpG site that is regulated by SNPs in the ASM list.

Information of ASM in human brain tissues was obtained from Zhang and colleagues [20]. Regulating SNPs within 1 Mb region of both ends of each CpG site were considered *cis*-acting, and all the other regulating SNPs were *trans*-acting. SNPs with region-wise p-value less than 0.05 in *cis*-acting and SNPs with genome-wide p-value less than 0.05 in *trans*-acting were selected in the ASM list in the present study. In total, we had 9,414 SNP-CpG pairs in autosomatic chromosomes, which included 9,042 *cis*-acting and 372 *trans*-acting pairs (In the Table S5 of the Zhang *et al*'s study). Figure 1 described our analysis flow-chart. To obtain gene level significance for BPD in the two GWA datasets, we first mapped SNPs to a gene region (using NCBI build 35 for the WTCCC and build 36 for the GAIN due to different genotyping platforms) if SNPs were located within 5 kb of both ends of the gene. In the WTCCC dataset, there were 193,837 SNPs mapped to 15,054 genes. Among these SNPs, 6,324 SNPs in the ASM list locate in 1,785 genes. In the GAIN dataset, there were 304,343 SNPs mapped to 16,385 genes, and 6,992 SNPs in the ASM list locate in 1,961 genes. We therefore divided genes into two sets, ASM and non-ASM set, in the subsequent pathway analysis. We defined the ASM set to include 1) genes with SNPs in the ASM list, and 2) genes with CpG site that is regulated by SNPs in the ASM list. Genes not in the ASM set were assigned into the non-ASM set. In total, we had 2,327 and 2,298 genes in the WTCCC and GAIN datasets, respectively.

The gene-wise p-value for each gene was defined by the most significant SNP within a gene region, which was commonly adopted in conducting pathway analysis [14]. For genes in the ASM set, the smallest p-value (*min-p*) among all SNPs in the ASM list in a gene region was used to represent the gene-wise statistic value. Similarly, for genes in the non-ASM set, the smallest p-value (*min-p*) of all SNPs in a gene region was used to represent the gene-wise statistic values. To account for potential bias caused by using minimum p-values to represent gene-wise statistic for genes with various sizes, we adopted the method by Yang and colleagues [23] to calculate normalized gene scores via 10,000 permutations for all the genes we analyzed. For each gene, a gene-size adjusted gene score was calculated and used in the following pathway analyses.

### Weighting procedure using the ASM information in pathways

To incorporate methylation information into GWA dataset in pathway-based analyses, we applied weighting procedures for ASM and non-ASM gene sets for each pathway. The overall proportion of significant SNPs (*i.e.* p-value<0.05) in the whole GWA dataset was first calculated (11.4% in the WTCCC and 10.2% in the GAIN datasets). We then compared the proportion of significant SNPs in a given gene with the average proportion of significant SNPs in the whole GWA dataset to evaluate whether a gene is more informative than average. We used Harmonic average (H) as the basis for weighting the average amount of informative genes in the ASM set and non-ASM set in a pathway. The weighting procedures are described below. For each pathway, the corresponding numbers in the ASM and non-ASM gene sets were *n* and *m*, respectively. *k_n_* and *k_m_* represent the numbers of informative genes in the two gene sets separately. Thus, the proportion of informative genes in the ASM and non-ASM sets was denoted as *k_n_/n* and *k_m_/m*. Assuming that R*_n_* and R*_m_* are two reduced fractions of k*_n_*/*n* and k*_m_*/*m*, the Harmonic average of R*_n_* and R*_m_*, H, was specified as 2/(1/R*_n_*+1/R*_m_*). The Harmonic average was chosen so that the average is less influenced by extreme R*_n_* or R*_m_*, values, and H was used to calculate the gene-wise weights for genes in the ASM and non-ASM sets in a pathway and to further reduce the potential bias in pathway analysis due to pathway size variation (*i.e.*, numbers of genes in each pathway vary). If the proportion of informative genes in the ASM set was greater than the non-ASM set, the weights for the ASM and non-ASM sets were R_n_/H and R_m_/H, respectively. If there were no informative genes in non-ASM set, we assigned a weight, ranging from 1 to 6, according to the proportion of informative genes (using 0.1, 0.3, 0.5, 0.7, and 0.9 as cutoff-values) to ASM set and 1 to non-ASM set. Otherwise genes in the ASM and non-ASM sets had equal weights.

### Statistical methods for pathway enrichment analysis

We downloaded canonical pathway information from Molecule Signature Database (MsigDB). The MsigDB consists of several available online sources of pathway databases and manually curated pathways from the literature, including Kyoto Encyclopedia of Genes and Genomes (KEGG), BioCarta, Reactome, Gene Ontology (GO) terms, and gene sets compiled from published biomedical literature [24], which listed 4,726 pathways and 22,429 genes. Pathways with extreme numbers of genes (*i.e.*, 10th percentile of pathway size distribution, less than 10 or more than 380) were removed from analysis to avoid stochastic bias or testing too general biological process. After mapping genes in GWA datasets into pathways, we tested in total 3,917 pathways for the WTCCC and 4,051 pathways for the GAIN datasets.

We applied both competitive and self-contained pathway analyses approaches [14]. Competitive method compares the statistics of genes in a given pathway with the rest of genes not in the pathway. Self-contained method compares the statistics of genes in a given pathway with the null genomic background [25]. To obtain more comprehensive information in pathway analyses, three statistical methods were performed to evaluate the enrichment of all pathways, Gene Set Enrichment Analysis (GSEA), sum-square-statistic and sum-statistic [26], [27], [28]. The details of calculation procedures were provided in our previous study [13]. In brief, GSEA method ordered a set of genes by the *min-p*, and the gene-wise statistic values (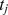) were defined as the chi-square statistic of the corresponding most-significant SNP. For each examined pathway, an enrichment score (ES) was calculated to evaluate association signals for all genes in the pathway. The sum-statistic or sum-square-statistic methods were to sum (the square) all gene-wise statistic values over the set of genes (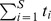 or 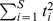) [28]. The three pathway-based approaches were analyzed with or without weighting procedures using the ASM information.

We performed five thousand permutations to obtain empirical p-values for each pathway. The Benjamini and Hochberg (BH) multiple comparison procedure was used to control for the false discovery rate (FDR) [29] in pathway analyses. A p-value less than 0.01 after FDR correction was considered significant in the present study. To examine the common processes or underlying biological themes among significant pathways, we also analyzed functions of genes in enriched pathways using GO terms, including domains in biological process, cellular component, and molecular function (http://www.broadinstitute.org/gsea/msigdb/annotate.jsp).

## Results

In the GAIN dataset, 88 pathways were significant, in which 32 were identified only by weighting the ASM information (Table S1). Similarly, we identified 15 pathways (11 of them were identified only when weighting procedure is applied for the ASM set) in the WTCCC dataset with p-value less than 0.01 after BH correction (Table S2). Among the 88 enriched pathways in the GAIN dataset, there were 32 (36.4%) from GO, nine (10.2%) from KEGG, and eight (9.1%) from Reactome. Among the 15 enriched pathways in the WTCCC dataset, there were six (40.0%) from GO and one (6.7%) from Biocarta. Three pathways consistently exhibited their biological relevance for BPD in both GWA datasets (Table 1). These 3 pathways were c*ation channel activity*, *gated channel activity*, and *metal ion transmembrane transporter activit*y. Additionally, the enriched pathways in the GAIN dataset (Table S1) were involved in a series of biological procedures and mechanisms, such as brain development and neuron function (e.g. *nervous system development*, *neurological system process*, *axon guidance*, etc.), component of extracellular matrix, ECM (*e.g. cell matrix adhesion, ECM receptor interaction, focal adhesion, integrin cell surface interactions*, *etc*.), neurotransmitter (*e.g. glutamate signaling pathway*), and ion channel activity (*e.g. potassium channel activity, voltage gated cation channel activity, calcium signaling pathway, etc.*). Most of the enriched GO pathways in the WTCCC dataset were associated with serotonin receptor and channel and transporter activity, such as *serotonin receptor activity*, *gated channel activity*, *cation channel activity*, and *metal ion transmembrane transporter activity* (Table S2).

**Table 1:** Concordant enriched pathways among GWA datasets of the GAIN and the WTCCC by different pathway-based methods.

Pathway name	Total genes in pathway	GAIN					WTCCC				
		No. of genes on list	% of ASM gene	Empirical p-value after the BH correction#			No. of genes on list	% of ASM gene	Empirical p-value after the BH correction#		
				GSEA	SUMST	SUMSQ			GSEA	SUMST	SUMSQ
GO_Cation channel activity	118	113	19.5	0.0881^a^	0.0000^c^	0.0386^b^	107	20.6	0.1741^a^	0.0000^b^	0.3656^b^
GO_Gated channel activity	121	114	19.3	0.0661^b^	0.0000^c^	0.0559^c^	109	22.0	0.0870^b^	0.0000^b^	0.2765^b^
GO_Metal ion transmembrane transporter activity	145	136	19.1	0.0939^b^	0.0000^c^	0.0000^a^	129	19.4	0.0870^b^	0.0000^b^	0.2765^b^

**ASM**: Gene set of allele-specific methylation; **GSEA**: Gene Set Enrichment Analysis; **SUMST**: sum-statistic; **SUMSQ**: sum-square-statistic.

# **:** The p-value after correction by the Benjamini and Hochberg (BH) multiple comparison procedure.

a **:** Empirical p-values of non-weighting method is less than weighting;

b : Empirical p-values of weighting method is less than non-weighting;

c : Empirical p-values of non-weighting and weighting are equivalent.

Using GO term analysis, we further examined genes in the enriched pathways of GAIN and WTCCC datasets (other than the original significant GO pathways) to search for common functions of these genes. In the GAIN datasets, there were 4,600 unique genes in 56 non-GO pathways. Table S3 shows the top 50 significant GO terms with p-value less than 0.05. Most of these significant GO terms were associated with cytoskeleton structure (*e.g. actin cytoskeleton organization and biogenesis*, *actin filament based movement*, etc.), ECM (*e.g. extracellular matrix structural constituent*, *collagen*, *integrin complex*, *integrin complex*, etc.), and cation and gated channel activity (*e.g. cation channel activity*, *nicotinic acetylcholine gated receptor channel complex*, *voltage gated calcium channel activity*, *etc*.). In the WTCCC datasets, there were 990 genes in 9 significant non-GO pathways. Table S4 exhibits the top 50 significant GO terms. Most of these significant GO terms were associated with ion channel activity (*e.g.*, *calcium, potassium, sodium, chloride channel activity*, etc.), transporter activity (*e.g. cation transmembrane transporter activity, inorganic cation transmembrane transporter activity*, etc.), and neurotransmitter receptor activitysuch as *serotonin receptor activity*. Table 2 displays the significant GO terms that were concordantly identified for BPD in both GWA datasets. The 29 GO terms were mainly associated with ion channel activity, such as *calcium channel*, *ligand gated channel*, *nicotinic acetylcholine gated channel*, *voltage gated channel*, etc.

**Table 2:** Concordant gene sets in the two GWA datasets of the GAIN and the WTCCC using Gene Ontology analysis.

Gene get name	NO. of gene in gene Set	GAIN (4,600 genes)		WTCCC (945 genes)	
		% of the overlap in Gene set	p-value*	% of the overlap in Gene set	p-value*
Calcium channel activity	33	90.9	2.31E−05	87.9	0.00E+00
Cation transmembrane transporter activity	211	78.2	5.39E−11	62.1	0.00E+00
Cation transport	146	65.8	1.88E−02	53.4	0.00E+00
Delayed rectifier potassium channel activity	12	100.0	1.18E−03	91.7	5.82E−10
Excitatory extracellular ligand gated ion channel activity	21	85.7	5.29E−03	81.0	4.91E−13
Extracellular ligand gated ion channel activity	21	85.7	5.29E−03	81.0	4.91E−13
Gated channel activity	121	86.0	5.18E−12	86.8	0.00E+00
Inward rectifier potassium channel activity	12	100.0	1.18E−03	91.7	5.82E−10
Ion channel activity	147	83.7	3.29E−12	82.3	0.00E+00
Ion transmembrane transporter activity	275	70.2	3.46E−06	53.8	0.00E+00
Ion transport	184	64.7	1.99E−02	47.8	0.00E+00
Ligand gated channel activity	39	79.5	2.81E−03	79.5	0.00E+00
Metal ion transmembrane transporter activity	145	86.9	4.33E−15	86.9	0.00E+00
Monovalent inorganic cation transport	93	69.9	7.16E−03	61.3	0.00E+00
Nicotinic acetylcholine activated cation selective channel activity	11	100.0	2.07E−03	81.8	1.78E−07
Nicotinic acetylcholine gated receptor channel complex	11	100.0	2.07E−03	81.8	1.78E−07
Potassium channel activity	50	96.0	4.29E−10	92.0	0.00E+00
Potassium ion transport	58	84.5	7.29E−06	77.6	0.00E+00
Sodium channel activity	17	82.4	2.72E−02	76.5	1.10E−09
Substrate specific channel activity	154	80.5	4.58E−10	78.6	0.00E+00
Substrate specific transmembrane transporter activity	341	67.5	3.72E−05	43.7	0.00E+00
Substrate specific transporter activity	388	63.4	5.25E−03	39.2	0.00E+00
Transmembrane transporter activity	371	66.6	7.59E−05	40.7	0.00E+00
Voltage gated calcium channel activity	18	94.4	5.88E−04	88.9	1.37E−13
Voltage gated calcium channel complex	15	93.3	2.69E−03	86.7	6.10E−11
Voltage gated cation channel activity	66	93.9	1.75E−11	90.9	0.00E+00
Voltage gated channel activity	73	90.4	3.53E−10	90.4	0.00E+00
Voltage gated potassium channel activity	36	100.0	1.57E−09	94.4	0.00E+00
Voltage gated potassium channel complex	40	90.0	5.76E−06	82.5	0.00E+00

**GAIN:** The analysis of biological gene sets by Gene Ontology was among 4,600 genes from 56 enriched pathways**; WTCCC:** The analysis of biological gene sets by Gene Ontology was among 945 genes from 9 enriched pathways.

We further identified genes that were over-represented in enriched pathways. We selected genes that were commonly involved in more than 20% out of all enriched pathways for each GWA dataset and had at least one SNP having *p*-value less than 0.05 in the GAIN or the WTCCC dataset, that is, more than 18 pathways in the GAIN and 3 pathways in the WTCCC datasets. The proportion of significant SNPs in these genes ranged from 1.2% to 54.5%. In total, there were 26 concordant genes that satisfied these criteria between the two GWA datasets (Table 3). They were mainly associated with calcium and potassium channel. In the WTCCC datasets, the over-represented genes were also associated with synaptic transmission (*e.g. ACCN1, CHRNAV6, HTR3B, HTR3A*), mediation of calcium ion release (*e.g. RYR1, RYR2, RYR3, TRPC3, TRPC4*), and channels of calcium, potassium, and sodium.

**Table 3:** Over-representing genes in enriched pathways in the two GWAS datasets of the GAIN and the WTCCC.

Gene	Set	GAIN			WTCCC		
		No. of SNP in gene	% of significant SNPs#	Smallest p-value	No. of SNP in gene	% of significant SNPs#	Smallest p-value
ACCN1	ASM	348	11.5	1.35E−03			
CACNA1A	ASM	48	2.1	4.06E−02			
CACNA1B	ASM	20	30.0	1.11E−02			
CACNA1C	ASM	205	3.4	3.82E−03	149	26.8	5.49E−05
CACNA1D	ASM	123	3.3	2.52E−02	68	14.7	4.45E−03
CACNA1E	ASM	45	6.7	3.92E−03			
CACNA2D1	non-ASM	77	2.6	2.47E−02			
CACNB2	ASM	185	11.9	5.07E−04	127	9.4	3.33E−05
CACNB3	ASM	1	100.0	3.63E−02			
CACNB4	ASM	68	1.5	4.59E−02	37	5.4	3.60E−02
CENPN	non-ASM	4	25.0	5.15E−22			
CHRNA6	non-ASM	6	33.3	2.84E−02			
HTR3B	non-ASM	14	7.1	6.88E−19			
KCNA2	ASM	5	20.0	4.85E−02			
KCNA4	non-ASM	5	20.0	3.64E−02			
KCNB2	ASM	142	4.9	2.62E−03	107	3.7	2.34E−04
KCNC1	ASM	11	54.5	1.59E−02	9	22.2	2.99E−02
KCNC4	ASM	10	10.0	1.60E−02			
KCND3	ASM	114	14.9	7.76E−04	59	5.1	1.31E−02
KCNE1	ASM	20	25.0	2.04E−02			
KCNG2	non-ASM	8	25.0	6.66E−04			
KCNH1	ASM	149	8.1	4.03E−03	79	3.8	1.19E−02
KCNH2	non-ASM	2	50.0	2.74E−02			
KCNJ1	non-ASM	11	9.1	3.95E−02			
KCNJ12					5	20.0	3.02E−02
KCNJ15					14	7.1	3.11E−02
KCNJ3	ASM	49	12.2	2.02E−03			
KCNJ5	non-ASM	18	5.6	7.22E−03			
KCNJ6	ASM	157	8.3	5.15E−03	97	2.1	2.12E−03
KCNK1	ASM	36	8.3	2.01E−02	21	38.1	3.04E−02
KCNK3					2	50.0	3.16E−05
KCNMB2	ASM	94	23.4	1.46E−03	63	11.1	1.03E−02
KCNN2					30	10.0	3.71E−03
KCNN3	ASM	83	15.7	4.28E−03	36	2.8	3.55E−02
KCNQ1	non-ASM	102	14.7	6.35E−04	64	4.7	2.53E−02
KCNQ3	non-ASM	162	1.2	1.65E−02	97	4.1	4.10E−03
KCNQ5					94	8.5	2.07E−03
KCNS1	non-ASM	5	40.0	6.14E−04	4	50.0	5.58E−05
KCNS3	ASM	21	14.3	3.18E−02	24	20.8	1.72E−02
P2RX4					3	33.3	2.93E−02
PKD2	ASM	27	22.2	7.65E−03	10	20.0	1.59E−02
RYR1					21	9.5	4.76E−03
RYR2					114	14.0	1.30E−03
RYR3					185	5.4	2.14E−03
SCN11A					20	30.0	1.13E−02
SCN2A					14	14.3	4.18E−02
SCN2B					11	18.2	7.79E−03
SCN5A					19	5.3	2.33E−02
SCN9A					22	9.1	2.08E−03
SERPINB5					21	4.8	2.98E−02
TRPC3					12	8.3	3.08E−02
TRPC4					57	10.5	8.55E−03

**ASM**: Gene set of allele-specific methylation; **Non-ASM:** Gene set of other than ASM in pathway analysis.

# **:** significant level: p-value less than 0.05.

## Discussion

For complex trait like bipolar disorder, the whole-genome screening provides comprehensive genetic data and pathway-based approaches offer complementary information to reveal underlying complex biological connections in the whole-genome scale. Results of pathway–based analysis not only can verify prior causal hypotheses for BPD (*e.g. neurotransmitter processes and neuron activity dysfunction in brain, etc*) but also to explore novel biological pathways [30], [31]. In the present study, we found enriched pathways for BPD to be related to ion channel activity such as calcium, potassium, and sodium ion. These findings are consistent with some of the presumed pathological mechanisms for BPD. Additionally, our results in the GAIN datasets suggest for the roles of extracellular matrix in brain to be involved in the development of bipolar disorder.

Among our reported significant pathways in the two datasets, some of them were identified through GO database, which include nervous system development, ion channel and transporter activity, extracellular matrix, *etc* in the GAIN dataset and gated channel activity in the WTCCC dataset. These enriched GO pathways for BPD are in line with findings from some of previous studies [32], [33], [34]. The other pathways we identified are mainly based on gene sets that were compiled from published biomedical literature (discussed later). We noted that few studies applying pathway based approaches for BPD utilized pathway sources only from KEGG or GO, which include only 25% to 40% genes in the whole human genome. Thus, a large proportion of genes with potential impacts for the trait of interest might be excluded from pathway analyses. This is clearly the restriction that results of pathway findings depend on the completeness and correctness of annotated pathway databases. Therefore, using more comprehensive pathway sources to include the whole genome (as we used in the present study) brings benefit to obtain more accurate and full understanding for the enriched pathways for bipolar disorder. A common phenomenon seeing in many GWA studies is that most of the reported genetic variants lack direct biological connection or knowledge (e.g. variants in a gene desert) [35], and are likely to be statistically artificial findings without meaningful biological explanations. The expectation of pathway analysis is to reveal more biological insights for the potential mechanisms of the trait, thus, to integrate other aspects of information from genetic regulation mechanisms into pathway analysis could be very useful. The present study focused on DNA methylation patterns, which represent as epigenetic markers to play critical roles in regulating gene expression. The commonly observed tissue specific allele-specific methylation could explain possible links between non-coding SNPs and gene function. We designed a weighting scheme to incorporate such information of functional variants into our pathway analyses for the GWA datasets of bipolar disorder. We found that several enriched pathways were only significant after applying the weighting scheme (36.3% in the GAIN dataset and 73.3% in the WTCCC datasets). These results with the weighting scheme reveal additional information, such as pathways related to exracellular matrix, long term potential, regulation of heart atrium and ventricle, methylated in acute lymphoblastic leukimia, etc in the GAIN dataset (Table S1) and gated and cation channel activity in the WTCCC dataset (Table S2). Prior studies have found that genes in some of these pathways are regulated by epigenetic mechanism. One recent study reported that epigenetic regulation of DNA demethylation of target genes, such as *RELN* (reelin, extracellular matrix serine protease) and *BDNF*, might underlie the mechanisms of synaptic plasticity and memory retention in rat medial prefrontal cortex [36] In addition, the expression of gene *SCN3A* that encodes for a subunit of voltage-gated sodium channel, which mainly expresses in the central nerve system, has found to be regulated by DNA methylation mechanism in mouse [37].

Some significant pathways in the present study identified through published biomedical databases were related to carcinogenesis (e.g. thyroid carcinoma, down-regulation of breast cancer, etc), specific targets of molecule complex (e.g. *NCAM1* interactions, targets of *CCND1* and *CDK4*, *CHREBP*, and *SEMA3B* etc.), and regulation of cellular processes or human diseases (e.g. arrhythmogenic right ventricular cardiomyopat, hypertrophic cardiomyopathyhcm and systolic heart failure, hematopoietic stem cell, etc). We examined the common functions of genes that included in these significant pathways to further explore their roles in the etiology of BPD. Results of GO term analysis in the GAIN dataset (Table S3) demonstrated the importance of collagen and extracellular matrix. Components of the extracellular matrix surround cell and mediate many important cellular processes such as cell differentiation, tissue rearrangement, and carcinogenesis. Neuron migration and colony from different brain areas which enrich the neuronal network with functional unit are highly associated with the extracellular matrix [38]. Disruption of this process in brain may be a potential cause of bipolar disorder. Interestingly, pathways that are related to cell movement (such as the extracellular matrix, focal adhesion, and regulation of actin cytoskeleton) were found to be associated with antipsychotic induced tremors in patient with mania episode [39]. In a GWA study of bipolar disorder, collagen type 11α2 (*COL11A2*), a component of extracellular matrix, was also shown to be associated with bipolar disorder comorbid with alcohol dependence [40]. This evidence altogether points to a new possibility to further investigate the roles of brain extracellular matrix in bipolar illness.

We also identified several important genes that over-represented in reported enriched pathways. Many of these genes are associated with different biological processes and functions, including synaptic transmission (*e.g. ACCN1, CHRNA6, HTR3B, HTR3A, etc*) and cation channels activity (*e.g. ACCN1, CACNA1C, KCNN3, etc)*. For genes encode for synapse components, prior association studies have indicated the involvement of certain genetic variants in a variety of psychiatric disorders [33], [41], [42], [43], [44], [45]. Genetic variants in genes related to serotonin transmission (*e.g. HTR1B*,*HTR3A, HTR5A, etc*) were associated with mood disorder [46]. Polymorphisms in *CHRNA6* (nicotinic alpha subunit 6 of neuronal cholinergic receptor), have also been reported to be associated with bipolar disorder [47].

In addition, many studies have reported that variations in gene *CACNAIC* (alpha 1C subunit of the L-type voltage-gated calcium channel) had strong association signals with bipolar illness [5]. Potassium channels are found in most cell types and control a wide variety of cell functions, such as regulation of action potential and resting membrane potential in neurons. Prior study reported that long repeats of *KCNN3* reduce potassium channel function and modify cognitive performance in schizophrenic patients [48]. *ACCN1* (neuronal amiloride-sensitive cation channel 1), a cation channel with high affinity for sodium, is also permeable for lithium and potassium ions. A recent genome-wide scan found that genetic variants in *ACCN1* were associated with response to lithium treatment in bipolar patients [49].

In conclusion, our study integrated methylation information with genome-wide genotyping data to bring biological insights into the underlying pathogenesis of bipolar illness. We identified significant pathways that are in line with evidence from prior causal hypotheses for bipolar disorder, and also reported novel biological pathways, such as the involvement of brain extracellular matrix in bipolar disorder. The strategy we applied provides another avenue to comprehensive our knowledge for the complex networks reside in the biological basis of bipolar disorder. Our findings could facilitate follow-up basic research to validate the functional and biological mechanisms for identified genes and pathways.

There are some limitations in the current study. First, the smallest p-value (as commonly adopted in other studies [14]) was used to define the gene-level statistic, thus, information of other markers in a gene region is excluded. Using a combined method to include all markers' information in a defined gene may provide slightly different results in pathway analysis, such as the Inverse Gamma method [50], random effects model, or Bayesian statistical methods [51]. In addition, the accuracy of pathway analysis results depends on the completeness and correctness of annotated pathway database. Although we have used the more comprehensive databases, there is still likely that some pathways were not included in our analysis. Second, we incorporated methylation information in brain tissues into pathway analysis, while other genomic information such as gene expression or other epigenetic regulation was not used. Integration of genomic information from different platforms may provide additional benefit to identify enriched pathways for bipolar disorder. Third, we used two major GWA datasets of BPD in Caucasian populations to obtain concordant findings. Although these are large-scale GWA datasets, the association results from meta- or mega- analysis can be used in the near future to further increase the power to uncover the underlying biological mechanisms for BPD.

## Supporting Information

Table S1 **88 Significant pathways in the GAIN dataset by pathway-based methods after correction for multiple comparisons.** #: The significant p-value after correction by the BH multiple comparison procedure; GSEA: Gene Set Enrichment Analysis; SUMSQ: sum-square-statistic; SUMST: sum-statistic.
(DOCX)

Table S2 **15 Significant pathways in the WTCCC dataset by pathway-based methods after correction for multiple comparisons.** #: The significant p-value after correction by the BH multiple comparison procedure; GSEA: Gene Set Enrichment Analysis; SUMSQ: sum-square-statistic; SUMST: sum-statistic.
(DOCX)

Table S3 **The top 50 significant GO terms with p-value less than 0.05 in the GAIN dataset.**
(DOCX)

Table S4 **The top 50 significant GO terms with p-value less than 0.05 in the WTCCC dataset.**
(DOCX)
