# Supplementary material for: Pathway Analysis Using Information from Allele-Specific Gene Methylation in Genome-Wide Association Studies for Bipolar Disorder
Source: PLoS One. 2013 Jan 9;8(1):e53092. doi: 10.1371/journal.pone.0053092 (PMC3541404; doi:10.1371/journal.pone.0053092)
Supplement: Table S1 — 88 Significant pathways in the GAIN dataset by pathway-based methods after correction for multiple comparisons. #: The significant p-value after correction by the BH multiple comparison procedure; GSEA: Gene Set Enrichment Analysis; SUMSQ: sum-square-statistic; SUMST: sum-statistic. (DOCX) [file pone.0053092.s001.docx]

**Supplementary Table S1. 88 Significant pathways in the GAIN dataset by pathway-based methods after correction for multiple comparisons**

| **Pathways** | **Significant P-values after BH-correction ^#^** | | | | | |
| --- | --- | --- | --- | --- | --- | --- |
|  |  | | | **With weighting scheme** | | |
|  | **GSEA** | **SQ** | **ST** | **GSEA** | **SQ** | **ST** |
| **GO** |  |  |  |  |  |  |
| Basement membrane |  | 0.0248 |  |  | 0.0097 |  |
| Cation channel activity |  | 0.0000 |  |  | 0.0000 | 0.0386 |
| Cation transmembrane transporter activity | 0.0000 | 0.0000 | 0.0000 | 0.0000 | 0.0000 | 0.0000 |
| Cation transport | 0.0000 | 0.0000 | 0.0000 | 0.0000 | 0.0000 | 0.0000 |
| Cell matrix adhesion |  | 0.0248 |  |  | 0.0000 |  |
| Cell substrate adhesion |  | 0.0248 |  |  | 0.0000 |  |
| Chromosome condensation |  |  |  |  | 0.0000 |  |
| Delayed rectifier potassium channel activity |  | 0.0365 |  |  | 0.0000 |  |
| Extracellular matrix |  | 0.0248 |  |  | 0.0000 |  |
| Extracellular matrix part |  | 0.0000 |  |  | 0.0000 |  |
| Gated channel activity |  | 0.0000 |  |  | 0.0000 |  |
| Glutamate signaling pathway |  | 0.0000 |  |  | 0.0000 |  |
| GTPase regulator activity |  | 0.0000 |  |  | 0.0000 |  |
| Guanyl nucleotide exchange factor activity |  | 0.0315 |  |  | 0.0097 |  |
| Integrin complex |  | 0.0248 |  |  | 0.0097 |  |
| Ion channel activity |  | 0.0121 |  |  | 0.0000 |  |
| Ion transmembrane transporter activity | 0.0000 | 0.0000 | 0.0000 | 0.0000 | 0.0000 | 0.0000 |
| Ion transport | 0.0000 | 0.0000 | 0.0000 | 0.0000 | 0.0000 | 0.0000 |
| Metal ion transmembrane transporter activity |  | 0.0000 | 0.0000 |  | 0.0000 | 0.0386 |
| Nervous system development |  | 0.0000 | 0.0386 | 0.0000 | 0.0000 | 0.0386 |
| Neurological system process | 0.0000 | 0.0000 | 0.0386 | 0.0000 | 0.0000 |  |
| Potassium channel activity |  | 0.0121 |  | 0.0000 | 0.0000 |  |
| Potassium ion transport |  | 0.0248 |  | 0.0352 | 0.0097 | 0.0000 |
| Proteinaceous extracellular matrix |  | 0.0248 |  |  | 0.0000 |  |
| Substrate specific channel activity |  | 0.0000 |  |  | 0.0000 |  |
| Substrate specific transmembrane transporter activity | 0.0000 | 0.0000 | 0.0000 | 0.0000 | 0.0000 | 0.0386 |
| Substrate specific transporter activity | 0.0000 | 0.0000 | 0.0000 | 0.0000 | 0.0000 |  |
| Transmembrane receptor protein phosphatase activity |  | 0.0000 |  |  | 0.0097 |  |
| Transmembrane transporter activity | 0.0000 | 0.0000 | 0.0386 | 0.0000 | 0.0000 |  |
| Voltage gated cation channel activity |  | 0.0409 |  |  | 0.0000 |  |
| Voltage gated channel activity |  |  |  |  | 0.0097 |  |
| Voltage gated potassium channel activity |  |  |  |  | 0.0097 |  |
| Voltage gated potassium channel complex | 0.0426 | 0.0000 |  | 0.0000 | 0.0000 |  |
| **KEGG** |  |  |  |  |  |  |
| Arrhythmogenic right ventricular cardiomyopat arvc |  | 0.0000 | 0.0000 | 0.0000 | 0.0000 | 0.0000 |
| Calcium signaling pathway |  | 0.0000 |  |  | 0.0097 |  |
| Dilated cardiomyopathy |  | 0.0000 | 0.0000 | 0.0491 | 0.0000 |  |
| ECM receptor interaction |  | 0.0000 | 0.0000 | 0.0352 | 0.0000 |  |
| Focal adhesion |  | 0.0000 |  |  | 0.0000 |  |
| Hypertrophic cardiomyopathy hcm |  | 0.0121 | 0.0000 |  | 0.0097 | 0.0386 |
| Long term potentiation |  | 0.0210 |  |  | 0.0097 |  |
| Pathways in cancer |  | 0.0210 |  | 0.0491 | 0.0097 |  |
| Vascular smooth muscle contraction |  | 0.0121 | 0.0386 |  | 0.0000 | 0.0000 |
| **REACTOME** |  |  |  |  |  |  |
| Axon guidance |  | 0.0000 |  |  | 0.0000 |  |
| Effects of PIP2 hydrolysis |  | 0.0000 | 0.0386 |  | 0.0000 | 0.0386 |
| Glucuronidation |  |  |  | 0.0000 | 0.0285 |  |
| Integrin cell surface interactions |  | 0.0000 |  |  | 0.0000 |  |
| NCAM1 interactions |  | 0.0000 |  |  | 0.0000 |  |
| RHO GTPase cycle |  | 0.0000 |  |  | 0.0000 | 0.0386 |
| Signalling by NGF |  | 0.0000 |  |  | 0.0000 |  |
| Transmembrane transport of small molecules |  | 0.0121 |  |  | 0.0097 |  |
| **Published biomedical literature** |  |  |  |  |  |  |
| Acevedo Liver cancer with H3K27ME3 UP | 0.0426 | 0.0000 |  |  | 0.0000 |  |
| Acevedo Liver cancer with H3K9ME3 UP |  | 0.0000 |  | 0.0491 | 0.0097 |  |
| Bertucci Medullary vs ductal breast cancer DN | 0.0000 | 0.0000 | 0.0000 | 0.0000 | 0.0000 | 0.0386 |
| Browne HCMV infection 24hr DN |  | 0.0000 |  |  | 0.0000 |  |
| Charafe Breast cancer basal vs mesenchymal DN |  | 0.0248 |  |  | 0.0097 |  |
| Dacosta UV response via ERCC3 TTD DN |  | 0.0000 |  | 0.0000 | 0.0000 |  |
| Dairkee TERT targets DN |  | 0.0248 |  |  | 0.0000 |  |
| Davicioni Molecular ARMS VS ERMS UP | 0.0426 | 0.0000 |  |  | 0.0000 |  |
| Delys Thyroid cancer DN |  | 0.0000 |  |  | 0.0000 |  |
| Firestein Proliferation | 0.0426 | 0.0121 |  | 0.0491 | 0.0000 |  |
| Hamai Apoptosis via trail UP |  | 0.0121 |  |  | 0.0000 |  |
| Horiuchi WTAP targets UP |  | 0.0000 |  |  | 0.0000 |  |
| Iwanaga Carcinogenesis by KRAS PTEN DN |  | 0.0121 |  |  | 0.0097 |  |
| Jaatinen Hematopoietic stem cell UP |  | 0.0000 | 0.0386 |  | 0.0000 | 0.0386 |
| Kaab Heart atrium vs ventricle DN |  | 0.0248 |  |  | 0.0000 |  |
| Lindgren Bladder cancer cluster 3 DN | 0.0000 | 0.0000 |  | 0.0000 | 0.0097 | 0.0386 |
| Manalo Hypoxia UP | 0.0000 | 0.0000 | 0.0000 | 0.0000 | 0.0000 | 0.0000 |
| Martinez Response to trabectedin |  | 0.0000 |  |  | 0.0000 | 0.0386 |
| Mohankumar TLX1 targets DN |  | 0.0121 |  |  | 0.0000 |  |
| Odonnell Metastasis UP |  | 0.0000 |  |  | 0.0000 | 0.0000 |
| Onder CDH1 targets 2 UP |  | 0.0000 | 0.0386 | 0.0491 | 0.0000 |  |
| Riggi EWING sarcoma progenitor UP | 0.0000 | 0.0000 |  | 0.0352 | 0.0000 |  |
| Rodrigues Thyroid carcinoma DN |  | 0.0210 |  |  | 0.0097 |  |
| Schuetz Breast cancer ductal invasive UP |  | 0.0000 |  |  | 0.0097 |  |
| Seitz Neoplastic transformation by 8p deletion DN |  | 0.0000 |  |  | 0.0097 |  |
| Senese HDAC1 targets DN |  | 0.0000 |  |  | 0.0097 |  |
| Siligan Bound by EWS FLT1 fusion |  | 0.0000 |  |  | 0.0097 |  |
| Siligan Targets of EWS FLI1 fusion DN |  |  | 0.0000 |  | 0.0000 | 0.0000 |
| Stark Prefrontal cortex 22q11 deletion UP | 0.0426 | 0.0000 |  | 0.0352 | 0.0171 |  |
| Takada Gastric cancer copy number DN |  | 0.0000 |  |  | 0.0097 |  |
| Takeda Targets of NUP98 HOXA9 fusion 6hr UP |  | 0.0000 |  |  | 0.0097 |  |
| Taylor Methylated in acute lymphoblastic leukemia | 0.0000 | 0.0315 |  | 0.0000 | 0.0386 |  |
| Vecchi Gastric cancer early DN |  | 0.0000 |  |  | 0.0000 |  |
| Verhaak AML with NPM1 mutated DN |  | 0.0121 |  |  | 0.0097 |  |
| Wang SMARCE1 targets UP |  | 0.0210 |  |  | 0.0000 |  |
| Yagi AML with 11q23 rearranged |  | 0.0248 |  |  | 0.0000 |  |
| Yauch Hedgehog signaling paracrine DN |  | 0.0248 |  |  | 0.0000 |  |

#: The significant p-value after correction by the BH multiple comparison procedure; **GSEA:** Gene Set Enrichment Analysis; **SUMSQ:** sum-square-statistic; **SUMST:** sum-statistic.
