# Supplementary material for: Pathway Analysis Using Information from Allele-Specific Gene Methylation in Genome-Wide Association Studies for Bipolar Disorder
Source: PLoS One. 2013 Jan 9;8(1):e53092. doi: 10.1371/journal.pone.0053092 (PMC3541404; doi:10.1371/journal.pone.0053092)
Supplement: Table S2 — 15 Significant pathways in the WTCCC dataset by pathway-based methods after correction for multiple comparisons. #: The significant p-value after correction by the BH multiple comparison procedure; GSEA: Gene Set Enrichment Analysis; SUMSQ: sum-square-statistic; SUMST: sum-statistic. (DOCX) [file pone.0053092.s002.docx]

**Supplementary Table S2. 15 Significant pathways in the WTCCC dataset by pathway-based methods after correction for multiple comparisons.**

| **Pathways** | **Significant P-values after BH-correction ^#^** | | | | | |
| --- | --- | --- | --- | --- | --- | --- |
|  |  | | | **With weighting scheme** | | |
|  | **GSEA** | **SQ** | **ST** | **GSEA** | **SQ** | **ST** |
| **BIOCARTA** CHREBP2 pathway |  |  |  | 0.0000 |  |  |
| **Go** |  |  |  |  |  |  |
| Ligand gated channel activity |  |  |  |  |  | 0.0000 |
| RNA helicase activity |  | 0.0000 |  |  | 0.0000 |  |
| Serotonin receptor activity |  |  |  |  |  | 0.0000 |
| Cation channel activity |  |  |  |  | 0.0000 |  |
| Gated channel activity |  |  |  |  | 0.0000 |  |
| Metal ion transmembrane transporter activity |  |  |  |  | 0.0000 |  |
| **Published biomedical literature** |  |  |  |  |  |  |
| Dorn Adenovirus infection 32hr DN |  | 0.0000 |  |  | 0.0000 |  |
| Dorn Adenovirus infection 48hr DN |  | 0.0000 |  |  | 0.0000 |  |
| Koyama SEMA3B targets DN |  |  |  |  | 0.0000 |  |
| Molenaar Targets of CCND1 and CDK4 DN |  |  |  | 0.0000 |  |  |
| Mueller Methylated in glioblastoma |  | 0.0000 |  |  |  |  |
| Provenzani Metastasis UP |  |  |  |  | 0.0000 | 0.0000 |
| Riggi EWING sarcoma progenitor UP |  |  |  | 0.0000 |  |  |
| Smid Breast cancer luminal A DN |  |  |  |  |  | 0.0000 |

#: The significant p-value after correction by the BH multiple comparison procedure; **GSEA:** Gene Set Enrichment Analysis; **SUMSQ:** sum-square-statistic; **SUMST:** sum-statistic.
