# Supplementary material for: Pathway Analysis Using Information from Allele-Specific Gene Methylation in Genome-Wide Association Studies for Bipolar Disorder
Source: PLoS One. 2013 Jan 9;8(1):e53092. doi: 10.1371/journal.pone.0053092 (PMC3541404; doi:10.1371/journal.pone.0053092)
Supplement: Table S3 — The top 50 significant GO terms with p-value less than 0.05 in the GAIN dataset. (DOCX) [file pone.0053092.s003.docx]

**Supplementary Table S3. The top 50 significant GO terms with p-value less than 0.05 in the GAIN dataset**

| **Gene Set Name** | **NO. of genes in gene Set** | **% of the overlap in gene set** | **p-value** |
| --- | --- | --- | --- |
| Cation channel activity | 118 | 93.2 | 0.00E+00 |
| Metal ion transmembrane transporter activity | 145 | 86.9 | 4.33E-15 |
| Ion channel activity | 147 | 83.7 | 3.29E-12 |
| Gated channel activity | 121 | 86.0 | 5.18E-12 |
| Voltage gated cation channel activity | 66 | 93.9 | 1.75E-11 |
| Cation transmembrane transporter activity | 211 | 78.2 | 5.39E-11 |
| Voltage gated channel activity | 73 | 90.4 | 3.53E-10 |
| Potassium channel activity | 50 | 96.0 | 4.29E-10 |
| Substrate specific channel activity | 154 | 80.5 | 4.58E-10 |
| Voltage gated potassium channel activity | 36 | 100.0 | 1.57E-09 |
| Ion transmembrane transporter activity | 275 | 70.2 | 3.46E-06 |
| Voltage gated potassium channel complex | 40 | 90.0 | 5.76E-06 |
| Potassium ion transport | 58 | 84.5 | 7.29E-06 |
| Calcium channel activity | 33 | 90.9 | 2.31E-05 |
| Substrate specific transmembrane transporter activity | 341 | 67.5 | 3.72E-05 |
| Collagen | 23 | 95.7 | 4.43E-05 |
| Transmembrane transporter activity | 371 | 66.6 | 7.59E-05 |
| Receptor complex | 56 | 80.4 | 2.00E-04 |
| Integrin complex | 19 | 94.7 | 3.52E-04 |
| Metal ion transport | 116 | 72.4 | 4.12E-04 |
| Extracellular matrix part | 57 | 79.0 | 4.27E-04 |
| Voltage gated calcium channel activity | 18 | 94.4 | 5.88E-04 |
| Delayed rectifier potassium channel activity | 12 | 100.0 | 1.18E-03 |
| Inward rectifier potassium channel activity | 12 | 100.0 | 1.18E-03 |
| Regulation of heart contraction | 24 | 87.5 | 1.44E-03 |
| Nicotinic acetylcholine activated cation selective channel activity | 11 | 100.0 | 2.07E-03 |
| Nicotinic acetylcholine gated receptor channel complex | 11 | 100.0 | 2.07E-03 |
| Sugar transmembrane transporter activity | 11 | 100.0 | 2.07E-03 |
| Voltage gated calcium channel complex | 15 | 93.3 | 2.69E-03 |
| Ligand gated channel activity | 39 | 79.5 | 2.81E-03 |
| Transmembrane receptor protein kinase activity | 51 | 76.5 | 3.03E-03 |
| Structural constituent of muscle | 32 | 81.3 | 3.54E-03 |
| Actin filament based movement | 10 | 100.0 | 3.63E-03 |
| Monosaccharide transmembrane transporter activity | 10 | 100.0 | 3.63E-03 |
| Substrate specific transporter activity | 388 | 63.4 | 5.25E-03 |
| Excitatory extracellular ligand gated ion channel activity | 21 | 85.7 | 5.29E-03 |
| Extracellular ligand gated ion channel activity | 21 | 85.7 | 5.29E-03 |
| Monovalent inorganic cation transport | 93 | 69.9 | 7.16E-03 |
| Amine transmembrane transporter activity | 41 | 75.6 | 1.06E-02 |
| Transmembrane receptor protein tyrosine kinase activity | 43 | 74.4 | 1.39E-02 |
| Cation transport | 146 | 65.8 | 1.88E-02 |
| Adp binding | 11 | 90.9 | 1.92E-02 |
| Ion transport | 184 | 64.7 | 1.99E-02 |
| Proteinaceous extracellular matrix | 98 | 67.4 | 2.32E-02 |
| Extracellular matrix | 100 | 67.0 | 2.61E-02 |
| Myoblast differentiation | 17 | 82.4 | 2.72E-02 |
| Sodium channel activity | 17 | 82.4 | 2.72E-02 |
| Amino acid transmembrane transporter activity | 29 | 75.9 | 2.86E-02 |
| Homeostasis of number of cells | 20 | 80.0 | 2.87E-02 |
| Skeletal muscle development | 31 | 74.2 | 3.75E-02 |
