# Supplementary material for: Pathway Analysis Using Information from Allele-Specific Gene Methylation in Genome-Wide Association Studies for Bipolar Disorder
Source: PLoS One. 2013 Jan 9;8(1):e53092. doi: 10.1371/journal.pone.0053092 (PMC3541404; doi:10.1371/journal.pone.0053092)
Supplement: Table S4 — The top 50 significant GO terms with p-value less than 0.05 in the WTCCC dataset. (DOCX) [file pone.0053092.s004.docx]

**Supplementary Table S4. The top 50 significant GO terms with p-value less than 0.05 in the WTCCC dataset**

| **Gene Set Name** | **NO. of genes in gene Set** | **% of the overlap in gene set** | **p-value** |
| --- | --- | --- | --- |
| Substrate specific transporter activity | 388 | 39.2 | 0.00E+00 |
| Transmembrane transporter activity | 371 | 40.7 | 0.00E+00 |
| Substrate specific transmembrane transporter activity | 341 | 43.7 | 0.00E+00 |
| Ion transport | 184 | 47.8 | 0.00E+00 |
| Cation transport | 146 | 53.4 | 0.00E+00 |
| Ion transmembrane transporter activity | 275 | 53.8 | 0.00E+00 |
| Monovalent inorganic cation transport | 93 | 61.3 | 0.00E+00 |
| Cation transmembrane transporter activity | 211 | 62.1 | 0.00E+00 |
| Metal ion transport | 116 | 64.7 | 0.00E+00 |
| Potassium ion transport | 58 | 77.6 | 0.00E+00 |
| Substrate specific channel activity | 154 | 78.6 | 0.00E+00 |
| Ligand gated channel activity | 39 | 79.5 | 0.00E+00 |
| Ion channel activity | 147 | 82.3 | 0.00E+00 |
| Voltage gated potassium channel complex | 40 | 82.5 | 0.00E+00 |
| Gated channel activity | 121 | 86.8 | 0.00E+00 |
| Metal ion transmembrane transporter activity | 145 | 86.9 | 0.00E+00 |
| Calcium channel activity | 33 | 87.9 | 0.00E+00 |
| Cation channel activity | 118 | 88.1 | 0.00E+00 |
| Voltage gated channel activity | 73 | 90.4 | 0.00E+00 |
| Voltage gated cation channel activity | 66 | 90.9 | 0.00E+00 |
| Potassium channel activity | 50 | 92.0 | 0.00E+00 |
| Voltage gated potassium channel activity | 36 | 94.4 | 0.00E+00 |
| Di _tri valent inorganic cation transmembrane transporter activity | 22 | 81.8 | 6.92E-14 |
| Voltage gated calcium channel activity | 18 | 88.9 | 1.37E-13 |
| Excitatory extracellular ligand gated ion channel activity | 21 | 81.0 | 4.91E-13 |
| Extracellular ligand gated ion channel activity | 21 | 81.0 | 4.91E-13 |
| RNA helicase activity | 24 | 75.0 | 1.01E-12 |
| Serotonin receptor activity | 11 | 100.0 | 5.43E-11 |
| Voltage gated calcium channel complex | 15 | 86.7 | 6.10E-11 |
| Di tri valent inorganic cation transport | 32 | 59.4 | 1.34E-10 |
| Delayed rectifier potassium channel activity | 12 | 91.7 | 5.82E-10 |
| Inward rectifier potassium channel activity | 12 | 91.7 | 5.82E-10 |
| Sodium channel activity | 17 | 76.5 | 1.10E-09 |
| RNA dependent atpase activity | 18 | 72.2 | 3.54E-09 |
| Calcium ion transport | 27 | 59.3 | 4.16E-09 |
| Calcium ion transmembrane transporter activity | 11 | 90.9 | 4.60E-09 |
| Voltage gated sodium channel activity | 11 | 90.9 | 4.60E-09 |
| Inorganic cation transmembrane transporter activity | 57 | 42.1 | 5.61E-09 |
| Plasma membrane part | 1141 | 16.9 | 1.13E-08 |
| Atp dependent rna helicase activity | 17 | 70.6 | 2.21E-08 |
| Integral to plasma membrane | 963 | 17.3 | 2.41E-08 |
| Intrinsic to plasma membrane | 977 | 17.2 | 4.03E-08 |
| Chloride channel activity | 19 | 63.2 | 1.44E-07 |
| Nicotinic acetylcholine activated cation selective channel activity | 11 | 81.8 | 1.78E-07 |
| Nicotinic acetylcholine gated receptor channel complex | 11 | 81.8 | 1.78E-07 |
| Plasma membrane | 1393 | 15.8 | 3.07E-07 |
| Anion channel activity | 20 | 60.0 | 3.22E-07 |
| Helicase activity | 49 | 38.8 | 1.02E-06 |
| ATP dependent helicase activity | 26 | 50.0 | 1.74E-06 |
| Protein phosphatase type 2a regulator activity | 14 | 64.3 | 4.64E-06 |
